# Multidisciplinary and standardized management of patients with delayed cerebral ischemia after aneurysmal subarachnoid hemorrhage

**DOI:** 10.1007/s00701-022-05347-y

**Published:** 2022-08-25

**Authors:** Amr Abdulazim, Carla Küppers, Katharina A. M. Hackenberg, Eva Neumaier-Probst, Mohamad Mansour Alzghloul, Jörg Krebs, Manfred Thiel, Hester Lingsma, Gabriel J. E. Rinkel, Christoph Groden, Nima Etminan

**Affiliations:** 1 Department of Neurosurgery, University Hospital Mannheim, Medical Faculty Mannheim, University of Heidelberg, Theodor-Kutzer-Ufer 1-3, 68167 Mannheim, Germany; 2 Department of Neuroradiology, University Hospital Mannheim, Medical Faculty Mannheim, University of Heidelberg, Mannheim, Germany; 3 Department of Anaesthesiology and Critical Care Medicine, University Hospital Mannheim, Medical Faculty Mannheim, University of Heidelberg, Mannheim, Germany; 4 Center for Medical Decision Sciences, Erasmus MC-University Medical Center Rotterdam, Rotterdam, the Netherlands

**Keywords:** Subarachnoid hemorrhage, Perfusion CT, Delayed cerebral ischemia, Vasospasm, Intra-arterial treatment

## Abstract

**Background:**

The appropriate management of delayed cerebral ischemia (DCI) after aneurysmal subarachnoid hemorrhage (aSAH) remains uncertain. We aimed to evaluate the effect of implementing a standardized protocol for detection and management of DCI after aSAH on cerebral infarction and functional outcome.

**Methods:**

We studied two cohorts of aSAH patients, one before (pre-implementation cohort: January 2012 to August 2014) and one after (post-implementation cohort: January 2016 to July 2018) implementation of a multidisciplinary approach, with standardized neurological and radiological assessment and risk-based medical treatment of DCI. We assessed the presence of new hypodensities on CT within 6 weeks after aSAH and categorized cerebral infarction into overall and DCI-related infarctions (hypodensities not within 48 h after IA repair and not attributable to aneurysm occlusion or intraparenchymal hematoma). Functional outcome was assessed at 3 months using the extended Glasgow outcome scale (eGOS), dichotomized into unfavorable (eGOS: 1–5) and favorable (eGOS: 6–8). We calculated odds ratios (OR) with corresponding 95% confidence intervals (CI’s), and adjusted for age, WFNS grade, Fisher score, and treatment modality (aOR).

**Results:**

In the post-implementation (*n* = 158) versus the pre-implementation (*n* = 143) cohort the rates for overall cerebral infarction were 29.1% vs 46.9% (aOR: 0.41 [0.24–0.69]), for DCI-related cerebral infarction 17.7% vs. 31.5% (aOR: 0.41 [0.23–0.76]), and for unfavorable functional outcome at 3 months 37.3% vs. 53.8% (aOR: 0.30 [0.17–0.54]). For patients with DCI, the rates for unfavorable functional outcomes at 3 months in the post-implementation versus the pre-implementation cohort were 42.3% vs. 77.8% (aOR: 0.1 [0.03–0.27]).

**Conclusions:**

A multidisciplinary approach with more frequent and standardized neurological assessment, standardized CT and CT perfusion monitoring, as well as tailored application of induced hypertension and invasive rescue therapy strategies, is associated with a significant reduction of cerebral infarction and unfavorable functional outcome after aneurysmal aSAH.

## Introduction

DCI is an important determinant for neurological outcomes after aSAH. Although it was traditionally assumed that DCI is predominantly caused by angiographic vasospasm, more recent studies highlighted that DCI is a multifactorial phenomenon, related to several pro-ischemic pathomechanisms [13]. This concept is supported by the fact that not all patients with vasospasm develop DCI, and not all patients who develop DCI have vasospasm.

To focus on cerebral hemodynamics instead of only angiographic vasospasm, CT perfusion imaging can be applied for detection and even prediction of DCI and may be especially useful in aSAH patients who are neurologically not assessable, but previous studies reported conflicting results in this respect [18]. For treatment of patients with clinical or radiological features of DCI, hypertension often is induced to ameliorate neurological worsening and prevent cerebral infarction and poor neurological outcomes [9, 16]. In patients with DCI refractory to induce hypertension, additional rescue therapy strategies, such as intra-arterial administration of nimodipine, are often considered [5, 7]. However, such treatments carry a risk of major complications and their risk–benefit ratio remains uncertain. We aimed to determine the effect of implementing a multidisciplinary approach, with frequent and standardized clinical assessment, standardized monitoring with CT perfusion, and a medical instead of an invasive initial step in the treatment of DCI on radiologically proven infarction and functional outcome in patients with aSAH.

## Methods

### Patient population

We studied one cohort before (pre-implementation cohort) and one after (post-implementation cohort) implementing a standardized management protocol (see below). We included all aSAH patients who underwent either surgical or endovascular intracranial aneurysm repair within 24 h after admission.

### Definitions

Clinical DCI was defined as a decrease in the level of consciousness (decrease in 2 points in the GCS) or an increase of 2 points in the National Institute of Health Stroke Scale or development of a new focal deficit lasting for at least 1 h and not explained by other factors (i.e., systemic complications and hydrocephalus) [17].

Radiological DCI was defined as a 1.5-fold prolongation of mean transit time (MTT) values, compared to baseline in serial CT perfusion imaging [3]. Severe angiographic vasospasm was defined as narrowing of the arterial diameter of > 70% from the baseline on digital subtraction angiography.

DCI-related cerebral infarctions were considered in the presence of new hypodensities within 6 weeks after the primary aSAH ictus but not within 48 h after IA repair and not attributable to surgical or endovascular treatment nor to intraparenchymal hematoma [17].

We regarded thromboembolic infarctions occurring during the intra-arterial catheter treatment with nimodipine and up to 48 h after catheter removal as nimodipine-catheter associated infarctions. Accordingly, any new intracranial hemorrhage during the intra-arterial nimodipine-catheter treatment with nimodipine was related to the necessary anticoagulation and thus, considered catheter associated.

Functional outcome was assessed at 3 months using dichotomized extended Glasgow outcome scale (eGOS 1–5: unfavorable and eGOS 6–8: favorable).

### DCI detection and management in the pre-implementation and post-implementation cohorts

**Fig. 1:**
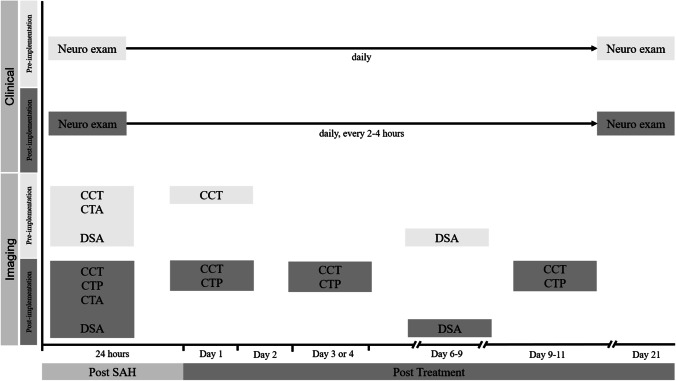
Clinical and radiological DCI monitoring and screening protocol. Abbreviations: CCT, cranial computed tomography; CTA, CT-angiography; DSA, digital subtraction angiography; i.a., intra-arterial; MTT, mean transit time; Neuro exam, neurological examination every 2 h; PCT, CT perfusion; SAH, subarachnoid hemorrhage

**Fig. 2:**
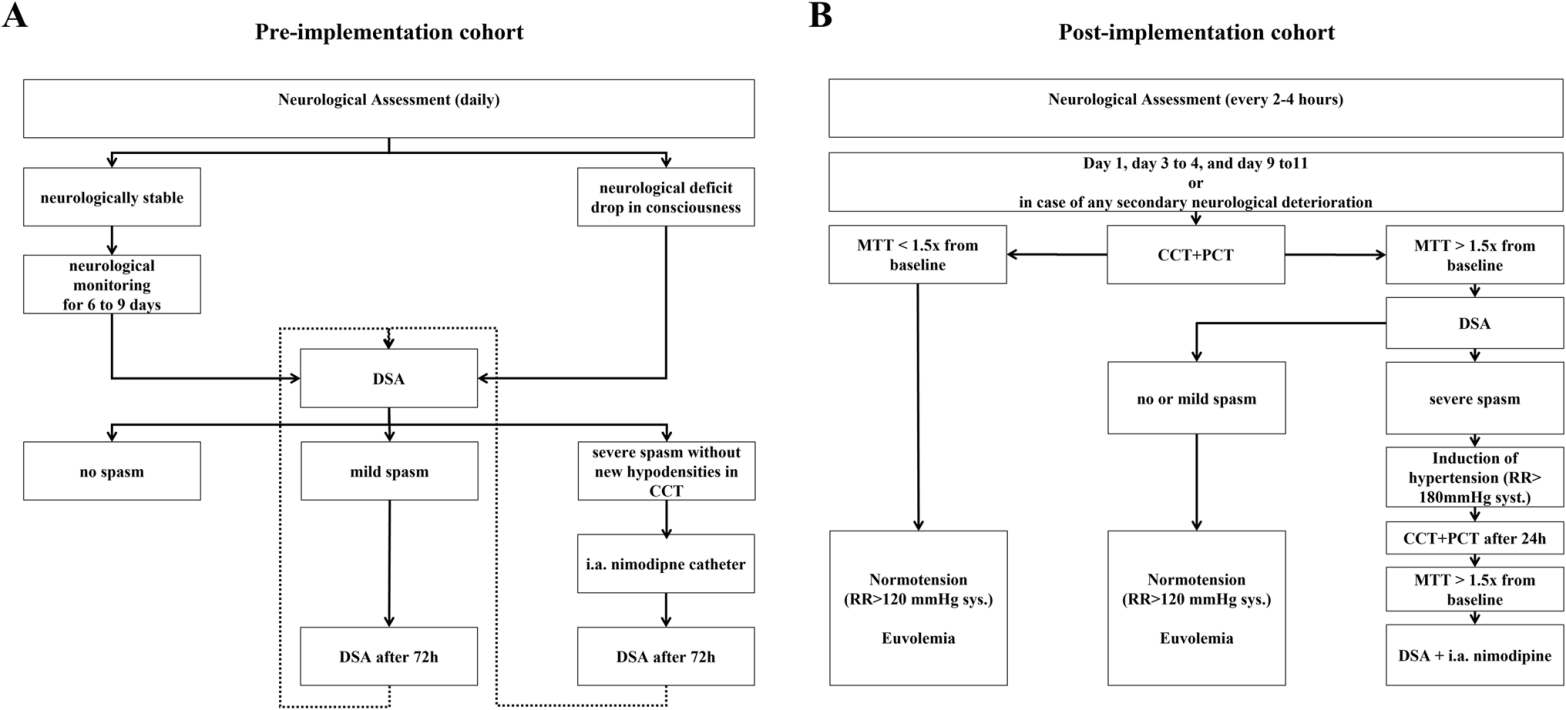
Algorithms for the management of patients with DCI. **A** DCI management algorithm in the pre-implementation cohort. **B** Escalating treatment protocol for patients with DCI in the post-implementation cohort. Abbreviations: CCT, cranial computed tomography; DSA, digital subtraction angiography; i.a., intra-arterial; MTT, mean transit time; PCT, perfusion computed tomography

Between January 2012 and August 2014 (pre-implementation cohort), there was no multidisciplinary consented protocol for the management of aSAH patients, who were admitted to our department. Management decisions were usually taken by the treating neurosurgeon or neuroradiologist on an individual basis, and patients were predominately treated according to the presence of angiographic vasospasm. In retrospect, the management in the pre-implementation cohort may be summarized as follows: Patients were (A) neurologically assessed in the intensive or intermediate care unit during daily rounds by neurosurgical residents and staff and (B) underwent digital subtraction angiography on days 6 to 9 after aneurysm repair or immediately upon clinical deterioration for the assessment of the presence of angiographic vasospasm (Figs. 1 and 2a). Clinical deterioration was defined as a new neurological deficit (aphasia and/or paresis) or a decline in consciousness. Angiographic vasospasm was regarded as clinically relevant in case of clinical deterioration suggestive of DCI or evidence of new hypodensities on unenhanced CT scans. Induced hypertension, if at all, was not consistently applied and was not part of a tailored and escalating treatment approach. Patients with severe, progressive, or clinically relevant vasospasm received an intra-arterial catheter for continuous nimodipine administration as first-line treatment. Angiographic vasospasm was monitored by a follow-up digital subtraction angiography after 72 h.

In fall 2015, the authors (NE, CG, ENP, JK) developed and implemented an interdisciplinary protocol for standardized detection and management of DCI (see below) in line with their previously reported studies [6, 10]. Following a transition period, from January 2016 onward, all aSAH patients admitted to our department (post-implementation cohort) were routinely discussed in a multidisciplinary team of neurosurgeons, neuroradiologists, and intensive care specialists, and management decisions in case of deterioration were taken based on the protocol and multidisciplinary discussion. The standardized detection protocol included thorough neurological examinations every 2–4 h by dedicated neurocritical care residents in combination with a novel CT perfusion screening protocol (Fig. 1). CT perfusion measurements were performed 6–12 h after aneurysm treatment, on days 3 or 4 as well as days 9 to 11 after aSAH, or in case of clinical features of DCI [6, 10, 17]. For patients who were and remained in perfect condition, the last CT perfusion was omitted. Additionally, digital subtraction angiographies were performed on admission and on days 6 to 9 after aSAH ictus and in case of clinical deterioration from DCI or CT perfusion deficits. In the case of persistent clinical signs of DCI, severe CT perfusion impairment or severe angiographic vasospasm, the patients in the post-implementation cohort were treated by means of a standardized, escalating treatment protocol: First, induced hypertension with a targeted systolic blood pressure of > 180 mmHg; second, solitary intra-arterial nimodipine bolus applications during catheter angiography; and third angiographic application of an intra-arterial catheter for continuous nimodipine administration over 48 h with CT perfusion imaging in between each escalating step (Fig. 2b).

The NEWTON-II study was a phase 3, multicenter, randomized, double-blind, placebo-controlled, parallel-group, efficacy and safety study comparing single-dose intraventricular nimodipine microparticles to oral nimodipine with respect to functional outcome and the incidence of DCI and cerebral infarction in patients after aSAH [2]. During this second phase, our center participated between August 2017 and March 2018 in this study, and patients admitted to our department during this time period were screened and if eligible enrolled and treated within the NEWTON-II study.

### Statistical analysis

Statistical analyses were performed using R version 3.6.2 [The R Foundation]. Continuous data were presented as mean (SD). Categorical data are presented as numbers (%). *P*-values for continuous data were derived from *t*-tests, and *P*-values for categorical data are derived from chi-square tests. Significance was defined at a level of *P* < 0.05. A logistic regression model was used to analyze the effects of the multidisciplinary and tailored escalating approach on functional and radiological outcomes. After calculation of crude odds ratios for functional and radiological outcomes, the model was adjusted for age, WFNS grade, Fisher score, and treatment modality.

## Results

We included 143 patients in the pre-implementation cohort and 158 in the post-implementation cohort (Table 1). In both the pre- and the post-implementation period, 9 patients died before IA repair and were therefore not included. Of the 158 patients in the post-implementation cohort, 8 participated in the NEWTON-II study, with equal distribution into the interventional and control arm [2]. In the pre-implementation cohort, 69 (48.3%) patients had angiographic vasospasm, of which 45 (31.5%) had clinically relevant vasospasms. Of those patients, 3 (4.3%) received induced hypertension, 2 (2.9%) single-shot, and 38 (55.1%) continuous intra-arterial nimodipine treatment (Table 1). In the post-implementation cohort, 126 (79.7%) patients developed any radiological DCI, of which 124 (98.4%) were treated by means of induced hypertension, 59 (46.8%) with single, and 14 (11.1%) with continuous intra-arterial nimodipine treatment. None of the patients in the pre- and post-implementation cohort developed renal complications. Especially, in view of the repeated CT perfusion imaging, we observed no renal failure associated with the regular use of contrast agents in the post-implementation cohort.

**Table 1:** Summary of patient characteristics as well as evidence for and treatment of DCI

	Pre-implementation cohort (*n* = 143)	Post-implementation cohort (*n* = 158)	*P*-value
*Patient characteristics*			
Age (mean; SD)	56.09 (11.23)	57.13 (11.67)	0.43
Female	87 (60.8%)	114 (72.2%)	0.05
Poor clinical condition on admission (WFNS grade 4–5)	47 (32.9%)	58 (36.7%)	0.56
Modified Fisher score 3–4 on admission	86 (59.4%)	126 (79.7%)	< 0.01
Aneurysm treatment modality			< 0.01
Surgical Endovascular	48 (33.6%) 95 (66.4%)	86 (54.5%) 72 (45.6%)	
*Evidence for and treatment of DCI*			
Clinical DCI	45 (31.5%)	70 (44.3%)	0.03
Radiological DCI	-	126 (79.7%)	
Angiographic vasospasm (> 70%)	69 (48.3%)	67 (42.4%)	0.37
Treatment of DCI			
Any treatment Induced hypertension i.a. nimodipine bolus Continuous i.a. nimodipine catheter	41 (59.4%) 3 (4.3%) 2 (2.9%) 38 (55.1%)	124 (98.4%) 124 (98.4%) 59 (46.8%) 14 (11.1%)	< 0.01 < 0.01 < 0.01 < 0.01

Abbreviations: *WFNS*, World Federation of Neurosurgical Societies; *DCI*, delayed cerebral ischemia; *i.a.*, intra-arterial

### Radiological and functional outcomes

**Fig. 3:**
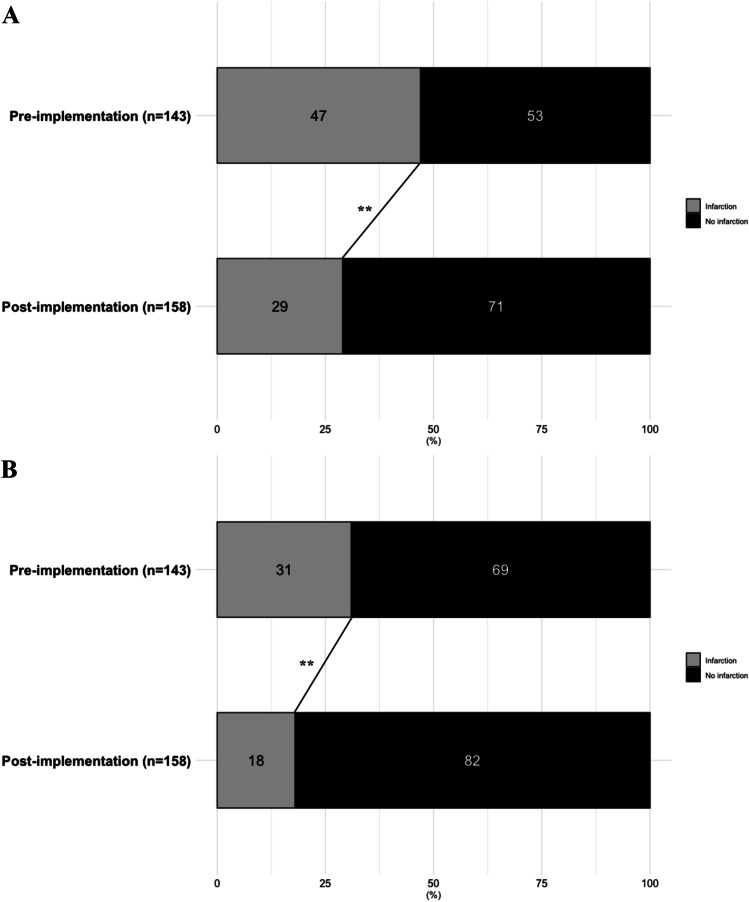
Stacked histograms of overall and DCI-related cerebral infarction. **A** Overall cerebral infarction rates after 6 weeks between the pre- and post-implementation cohorts. **B** DCI-related cerebral infarction rates after 6 weeks between the pre- and post-implementation cohorts. **P < 0.01

Any cerebral infarction occurred in 67 (46.9%) patients in the pre-implementation cohort and in 46 (29.1%) in the post-implementation cohort (aOR: 0.41 [0.24–0.69]). DCI-related cerebral infarction occurred in 45 (31.5%) patients in the pre-implementation cohort and in 28 (17.7%) patients in the post-implementation cohort, aOR was 0.41 [0.23–0.76] (Table 2, Figs. 3 and 5).

**Table 2:** Radiological and functional outcomes

	Pre-implementation cohort (*n* = 143)	Post-implementation cohort (*n* = 158)	aOR* (95% CI)
*Radiological outcome*			
Overall infarction within 6 weeks	67 (46.9%)	46 (29.1%)	0.41 (0.24–0.69)
DCI-related infarction within 6 weeks	45 (31.5%)	28 (17.7%)	0.41 (0.23–0.76)
Nimodipine-catheter associated infarction Nimodipine-catheter associated hemorrhages	4 (10.5%) 4 (10.5%)	1 (7.1%) -	- -
*Functional outcome in all patients*			
Poor outcome at 3 months	77 (53.8%)	57 (37.3%)	0.30 (0.17–0.54)
*Functional outcome in patients with DCI*			
Poor outcome at 3 months	35 (77.8%)	52 (42.3%)	0.10 (0.03–0.27)

Abbreviations: *aOR*, adjusted odds ratio; *95% CI*, confidence interval; *DCI*, delayed cerebral ischemia

^*^Adjustment for age, WFNS, Fisher, and treatment modality

Nimodipine-catheter-associated infarctions in the pre-implementation cohort versus the post-implementation cohort occurred in 4 (10.5%) patients vs. 1 (7.1%) patient.

**Fig. 4:**
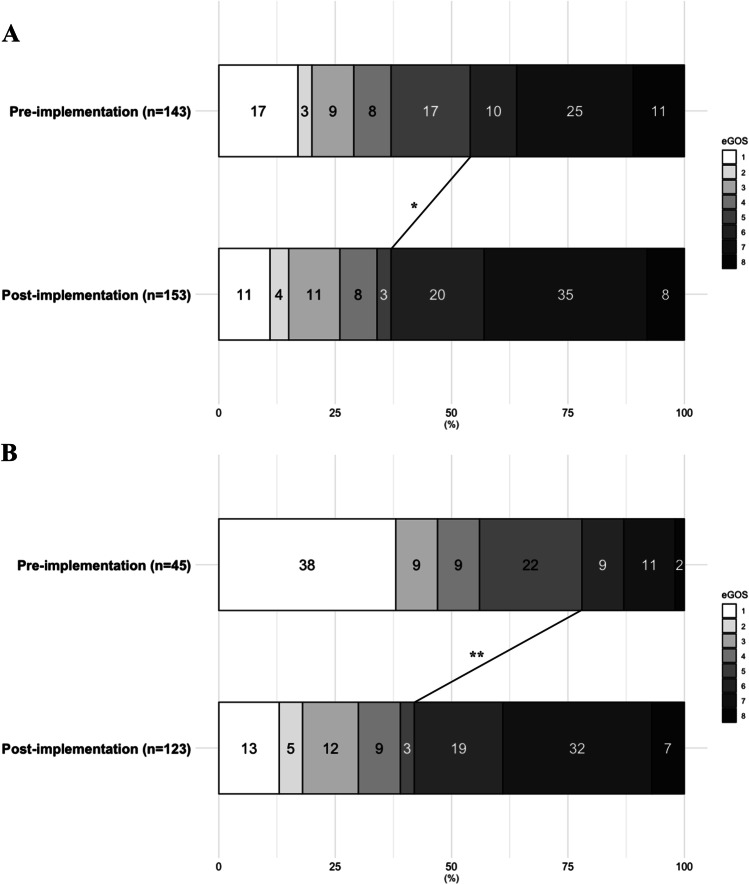
Stacked histograms of functional outcome. **A** Overall functional outcome at 3 months. **B** Functional outcome in patients with DCI at 3 months. Distribution of scores according to the extended Glasgow Outcome Scale between the pre-implementation cohort (January 2012–August 2014) and the post-implementation cohort (January 2016– July 2018) for patients with DCI. Abbreviations: eGOS, extended Glasgow Outcome Scale. *P < 0.05**;** **P < 0.01

**Fig. 5:**
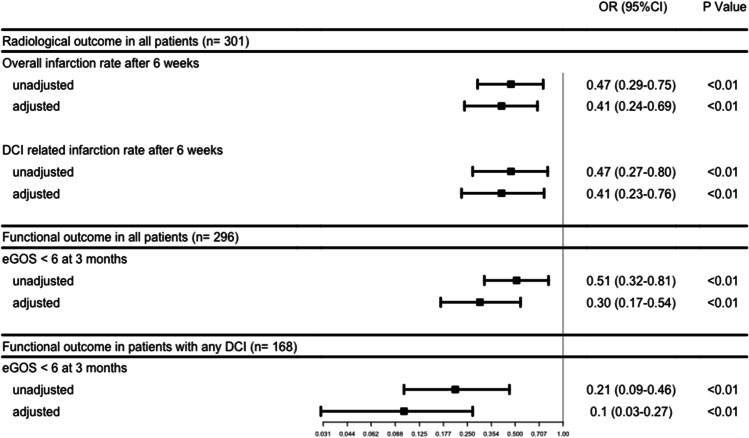
Effect of standardization on radiological and functional outcome. Abbreviations: OR, odds ratio; 95% CI, 95% confidence interval; DCI, delayed cerebral ischemia; eGOS, extended Glasgow Outcome Scale. Pre-implementation cohort as reference. Adjustment for age, WFNS, Fisher, and treatment modality

In the pre-implementation cohort, 77 (53.8%) patients had an unfavorable functional outcome versus 57 (37.3%) in the post-implementation cohort (aOR: 0.30 [0.17–0.54]) (Table 2, Figs. 4a and 5).

In patients who developed DCI, in the pre-implementation cohort, unfavorable outcome was present in 35 (77.8%) patients, compared to 52 (42.3%) patients in the post-implementation cohort (aOR: 0.1 [0.03–0.27]) (Table 2, Figs. 4b and 5).

The exclusion of the 8 patients who were enrolled in the NEWTON-II trial from the analysis did not show any effect on the results (Table 3).

**Table 3:** Radiological and functional outcomes before and after excluding NEWTON-II patients

	aOR* (95%CI) incl. NEWTON II patients	aOR* (95%CI) excl. NEWTON II patients
*Radiological outcome*		
Overall infarction within 6 weeks	0.41 (0.24–0.69)	0.40 (0.23–0.69)
DCI-related infarction within 6 weeks	0.41 (0.23–0.76)	0.42 (0.23–0.76)
*Functional outcome in all patients*		
Poor outcome at 3 months	0.30 (0.17–0.54)	0.30 (0.17–0.55)
*Functional outcome in patients with DCI*		
Poor outcome at 3 months	0.10 (0.03–0.27)	0.10 (0.03–0.28)

Abbreviations: *aOR*, adjusted odds ratio; *95% CI*, confidence interval; *DCI*, delayed cerebral ischemia

^*^Adjustment for age, WFNS, Fisher, and treatment modality

### Effect of DCI treatment in patients with exclusive radiological DCI

**Fig. 6:**
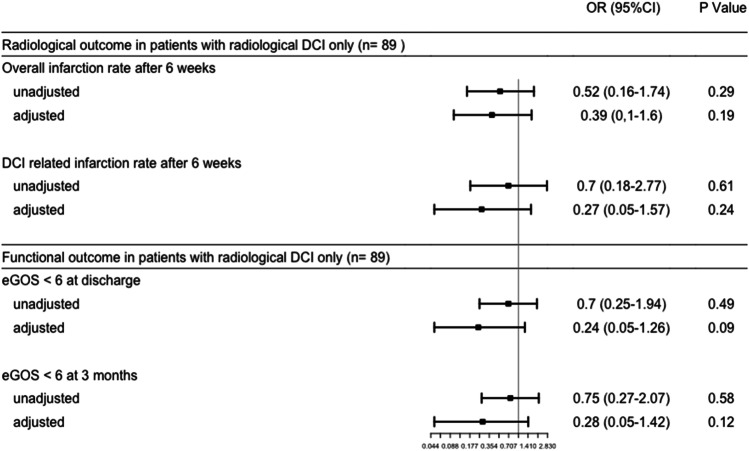
Effect of increased monitoring with CT perfusion. Abbreviations: OR, odds ratio; 95% CI, 95% confidence interval; DCI, delayed cerebral ischemia; eGOS, extended Glasgow Outcome Scale. Pre-implementation cohort as reference. Adjustment for age, WFNS, Fisher, and treatment modality

Of all patients in the post-implementation cohort who received DCI treatment, 24 patients were treated based on radiological DCI only and exclusively received induced hypertension. Compared to patients in the pre-implementation cohort with no evidence of clinical DCI or hemodynamically relevant macrovascular spasms, the rates of overall and DCI-related cerebral infarction at 6 weeks after SAH ictus were lower in the post-implementation cohort, which was more distinct after adjustment for age, WFNS grade, Fisher score, and treatment modality (Fig. 6). These patients tended to have lower rates of unfavorable functional outcomes at discharge as well as at 3 months. This effect was more pronounced after adjustment for age, WFNS grade, Fisher score, and treatment modality (Fig. 6).

## Discussion

Our study revealed that a multidisciplinary approach, with frequent and standardized clinical assessment, standardized monitoring with CT perfusion, and staged and escalating management instead of invasive primary step in treatment in case of DCI was associated with a significantly improved radiological and functional outcome after aSAH, compared to aSAH patients who were, in the absence of a dedicated multidisciplinary standard operating procedure.

Previous studies reporting conflicting results on the role of DCI detection and/or rescue therapy after SAH: A study on multimodal and invasive monitoring for DCI and a prespecified algorithm for endovascular rescue treatment (transluminal balloon angioplasty and/or continuous intraarterial nimodipine) reported good functional outcomes at 3 months in 35.5% in those patients who received endovascular rescue treatment [19]. Our protocol did not only yield a lower rate of patients requiring intra-arterial catheter treatment (11.1% vs. 20.1%) but also a higher rate of favorable functional outcomes in the subgroup of patients in the post-implementation cohort who received continuous intra-arterial nimodipine treatment (57.1% vs. 35.5%) [19].

Another study on frequent and early endovascular treatment of angiographic vasospasm reported a reduced incidence of DCI-related cerebral infarction and an improved functional outcome [12]. However, the reported rates of DCI-related infarction (20.8%) and unfavorable outcomes (defined as mRS > 2, 44%) in this study were higher than those in our post-implementation cohort (17.7% for cerebral infarction and 34% for the unfavorable outcome, considering a comparable unfavorable outcome cutoff at eGOS < 5).

The improved outcome in the post-implementation cohort may be explained by the strict multidisciplinary approach, the regular clinical assessment by dedicated personnel, the frequent CTP measurements, or the more stepwise approach in patients with DCI compared to the pre-implementation cohort, or a combination of these factors. More vigilant multidisciplinary management may in itself result in better outcomes, independent of specific interventions used in the protocol [15]. Additionally, the implementation of a standardized protocol may lead to a higher dedication of the treating team in terms of a more thorough clinical and neurological monitoring. Alternatively, the radiological surveillance implemented in our protocol for detection of DCI using serial CT perfusion imaging, especially in patients who are clinically not assessable, may have also resulted in earlier detection of DCI and thereby earlier rescue therapy for DCI. This is supported by previous CT perfusion studies in the setting of aSAH, since CT perfusion may detect both macro- and microcirculatory disturbances prior to clinical manifestation of DCI [4, 18]. Furthermore, a risk-based, escalating rescue therapy in patients with clinical and/or radiological DCI may have resulted in a beneficial risk–benefit ratio and thereby improved outcomes, compared to sole angiographic vasodilation as a first-line therapy [7]. Although the benefit of induced hypertension for the management of DCI after SAH is unproven, [8] it carries a risk of complications. Moreover, intra-arterial treatment strategies harbor an additional risk for new cerebral infarcts due to thromboembolism and arterial dissection [1]. The higher Fisher score may partly explain the higher rate of clinical DCI in the post-implementation cohort. Nevertheless, in view of the better outcome in the post-implementation cohort in our study compared to standardized management protocols of previous studies, it seems more likely that not only the protocol implementation itself but especially the diagnostic and therapeutic means in our protocol may have had a meaningful effect on radiological and functional outcome [12, 19].

We acknowledge several limitations: First, our study comprises a retrospective analysis of prospectively collected data and one could argue that our findings are no more than a “self-fulfilling prophecy.” Second, as our protocol essentially comprises multiple interacting “interventions,” we cannot further analyze the individual contribution of each of these components to the outcomes. Third, patients in the post-implementation cohort may have higher risks to develop DCI and poor neurological outcomes. This is partly reflected in the higher rate of clinical DCI in the post-implementation cohort. We addressed this by adjusting our logistic regression models for these factors, which resulted in an even more pronounced effect toward improved radiological and functional outcomes in the post-implementation cohort. Fourth, it is often debated whether the radiation exposure of serial CT perfusion imaging and repeated patient transports are justified to improve outcomes in aSAH patients. In line with previous studies and in view of the absolute risk reduction for cerebral infarction and unfavorable outcome in our data, our study indicates a reasonable risk–benefit ratio [11, 14]. Additionally, the inclusion of patients treated within the NEWTON-II trial may have seemed to introduce some bias in the post-implementation cohort with respect to functional outcomes. However, our exploratory analyses without these 8 patients did not reveal any effect on our results, which is most likely explained by the equal distribution of patients into the two treatment arms and especially because the NEWTON-II trial protocol complied with our own institutional standard. Since these patients were treated in accordance with our standardized protocol described here, we refrained from omitting these patients from the complete dataset. Lastly, we cannot entirely rule out the effect of improved outcome after aSAH due to advances in aSAH management in general over time. However, since the two observational periods differed only by 3 years, we do not foresee time trends in aSAH outcome as a major contributor to our findings.

## Conclusion

Our data highlight that protocol-based, rigorous detection and management strategies for DCI may reduce the rate of unnecessary invasive rescue procedures and, consequently, complication rates. To avoid excessive use of CTP, it is important to tailor the indication for CTP imaging to the risk of DCI and/or neurological status of SAH patients. A cluster randomized trial may provide more definitive data on the associations we found.
